# Effects of mindfulness on depression in college students: mediating role of psychological resilience and moderating role of gender

**DOI:** 10.1186/s40359-023-01468-w

**Published:** 2024-01-16

**Authors:** Junliang Zhang, Shuang Zheng, Zhongzheng Hu, Jingming Wang

**Affiliations:** Science and Technology College, Nanchang Hangkong University, Jiujiang, China

**Keywords:** Mindfulness, Depression, Psychological resilience, College students, Gender

## Abstract

**Objective:**

A questionnaire was administered to 936 college students to investigate the mediating effect of psychological resilience in the association between mindfulness and college student depression, as well as the moderating role of gender.

**Methods:**

For this study, data was collected between 20 April and 1 May 2023 at three universities in Jiangxi Province using a random sampling method. 963 Chinese university students were surveyed using the Adolescent Mindfulness Scale, the Psychological Resilience Scale, and the Depression Self-Rating Scale.SPSS24.0 software was used for correlation analysis and linear regression analysis, and PROCESS v3.4 model 7 was used to analyze this mediated model with moderating effects.

**Results:**

In the mediated effects model, the direct effect of mindfulness on college depression levels was significant (95% CI -0.43, -0.31); the indirect effect of mindfulness on college depression through psychological resilience was also significant (95% CI -0.34, -0.23); the interaction effect of mindfulness and gender was also found to be significant (95% CI 0.03, 0.16) in the mediated model with moderation.

**Conclusion:**

Mindfulness not only affect depression directly, but also indirectly through the mediating effect of psychological resilience. At the same time, the prediction of psychological resilience by mindfulness was also moderated by gender, with girls' psychological resilience being more affected by the level of mindfulness compared to boys.

## Introduction

College students' mental health issues have become increasingly serious over the past few years, attracting more attention. At the same time, it has also been found that depression and anxiety are common psychological problems in this group [1], especially when students in a constant state of anxiety and depression eventually become depressed [2]. Depression often manifests itself in the form of lack of motivation, reduced will, sleep disturbance, and low academic efficiency, and in severe cases, even self-harm and suicidal ideation or behavior [3]. According to the American College Health Association, the rate of college students diagnosed with depression has exceeded 15% since 2008 [4] and college students are gradually becoming more susceptible to suicide [5]. Depression among college students has been increasing over the past decade [6]. In China, the current depression detection rate among college students is also as high as 31.35% and is on the rise [7], therefore it has become particularly important to seek the causal mechanisms or methods to prevent and address depression problems. In this article, we explored psychological resilience as a possible mediator of mindfulness's effect on depression and gender.

Mindfulness is an attribute of consciousness that originated in Eastern Buddhism and enhances physical and mental health [8], and is defined as non-judgmental, intentional attention and awareness of the here and now [9]. The principles and methods of mindfulness were first applied in practice in 1979 [10] and are now widely used worldwide as a method for maintaining mental health [11]. In addition to being a skill, mindfulness is a way of life [12]. Mindfulness has a strong therapeutic effect on emotional well-being, and the higher the level of mindfulness, the less distress an individual experiences [13].

Due to its ability to increase emotion and attention regulation through awareness,stress, depression, and subjective well-being have been positively influenced by mindfulness in adolescents [14]. Research has also shown that interventions based on mindfulness are effective for emotional and behavioral regulation in adolescents, benefiting pro-social behavior and mental health, reducing anxiety and depression symptoms, for example [15]. It has also been suggested that mindfulness exercises can be used as a means of preventing depressive mood while increasing college students' mindfulness [16]. Some related studies even found that students' depressive moods were lower when they were mindful [17]. That is, a high level of mindfulness in college students allows them to focus positively on the problem they're facing and on the problem itself, enabling individuals to disengage from negative emotions and avoid depressive moods. It has also been found that after a period of mindfulness practice, college students in a depressed state can divert their attention from events that cause negative emotions and actively regulate their negative emotions, which can help reduce depression and anxiety [18]. In summary, this study hypothesized that mindfulness negatively predicted depression in college students.

### The mediation of psychological resilience

Psychological resilience refers to one's ability to adapt to challenges and adverse events [19], such as trauma, threats, or other significant stressors. In addition, it is a defense mechanism for thriving in adversity [20] and preventing depression [21]. Individuals with low psychological resilience are more likely to be unable to cope with adversity and thus react pathologically, while people who are strong psychological resilience escape it more easily [22].

Mindfulness, an important element of psychological protection, not only enhances levels of psychological resilience, but also significantly and positively predicts psychological resilience [23]. A study of mindfulness interventions with college students found that mindfulness practice not only increased levels of psychological resilience but also correspondingly improved their depression [9]. A foreign meta-analytic study found that trait mindfulness can positively and positively predict psychological resilience [24], while mindfulness training can also enhance the level of psychological resilience [25]. By testing 431 Chinese medical students, Xu Xin et al. [26] found that mindfulness helped medical students better cope with stress and improve depression by enhancing their levels of psychological resilience. It has also been demonstrated that psychological resilience may also be beneficial for individuals struggling with depression, anxiety and other emotional disorders [27]. In this study, psychological resilience was hypothesized to be a mediating factor between mindfulness and depressed mood in college students.

### The moderation of gender

The study of mediating effects can reveal the mechanism of action how variables interact, while moderation effects can reveal what influences a relationship's strength and direction. Moderating effects is an important complement to the exploration of mediating effects. The mediation analysis of mindfulness on college students' depression helps to understand the process by which mindfulness exert influence on college students' depression through psychological resilience, but this process may be influenced by gender differences, i.e., there are differences between males and females with respect to mindfulness that have different effects on college students' psychological resilience, thus indirectly affecting college students' depression. According to Kang et al. [28], adolescents' psychological resilience might be affected differently by mindfulness depending on their gender. Girls had a more significant increase in emotional well-being and benefited more from the effects of the mindfulness intervention compared to boys.Gender role theory suggests that social role expectations and personality traits influence the psychological and behavioral characteristics of individuals of different genders [29], and thus the roles and responsibilities held naturally differ in psychological and behavioral performance [30]. Gender differences have been found in previous studies on mindfulness and psychological resilience, with female college students scoring significantly higher on mindfulness measures than males [31], possibly due to the fact that females have higher levels of psychological resilience due to their higher susceptibility to mindfulness than males. Therefore, girls may be more susceptible to their own level of mindfulness in terms of perceived psychological resilience compared to boys. However, it has also been shown that college students who are male are more mindful than those who are female [32], that is to say,female students with stronger levels of positive thinking showed better psychological resilience.We hypothesized that gender might moderate the first half of the pathway of mindfulness, psychological resilience, and depression.

### The present study

Mindfulness may be one of the most important predictors of depressed mood among college students, so how mindfulness influence depressed mood among college students? The underlying mechanisms investigated are inadequate, and little is known from previous research. Existing research suggests that mindfulness has a positive effect on depression, for example, positive psychology takes a perspective to explain that positive resources and volition can act as protective factors to reduce the incidence of depression. Our study investigated the mediating effect of psychological resilience in the association between mindfulness and college student depression, as well as the moderating role of gender. We hypothesized that psychological resilience would mediate the association between mindfulness and depression among college students. Gender might moderate the first half of the pathway of mindfulness, psychological resilience, and depression.

### Study hypothesis

Our study focused on the link between mindfulness and depression among college students, as well as the internal mechanisms at work. Mindfulness, psychological resilience and depression among college students were measured in an online questionnaire. Based on these research objectives, we formulated 3 hypotheses: (1) H1: Mindfulness has a significant predictive effect on depression in college students. (2) H2: Psychological resilience is a mediator between mindfulness and depression in college students. (3) H3:gender might moderate between mindfulness and psychological resilience.

**Fig. 1:**
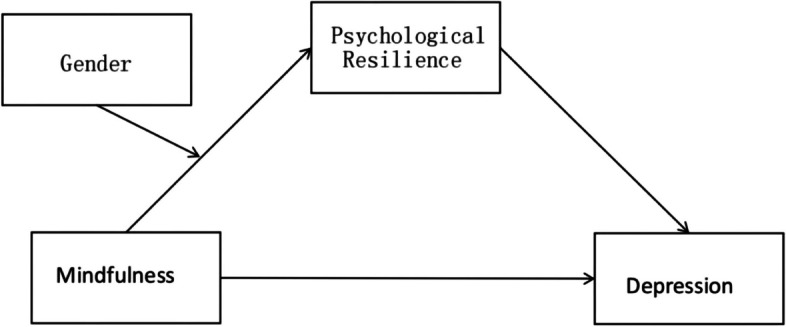
Hypothesis testing model

In summary, a substantial percentage of college students struggle with depression, and an attempt to find and discover some factors and their mechanisms that can better resist and reduce the risk of depression from the perspective of positive psychology may provide practical references and guidance for the prevention and intervention related to college students' mental health problems. In order to comprehensively explore the effects of mindfulness on college students' depression levels and its mechanisms, this paper aims to build a moderated mediation model based on previous studies (See Fig. 1).

## Methods and instruments

### Subjects

College students from three universities in Jiangxi Province, China were recruited using a stratified sampling method between April 20 and May 1, 2023. The recruited students were freshmen to seniors, and all participants were asked to complete the questionnaire anonymously on Questionnaire Star (a smartphone and web-based questionnaire platform). The questionnaire was completed by 936 of the 1000 college students recruited for this study, yielding a valid response rate of 93.6%. The average age of the university students who took part in the test was 20 ± 1.96, with 512 male students (54.71%) and 424 female students (45.29%). The trial was approved in advance by members of the Academic Committee of Science and Technology College, Nanchang Hangkong University, and every subject signed an informed consent form.

The questionnaire for this study used a version that has been used and widely proven, and its validity and reliability are not in question. Also, following its administration, we evaluated the questionnaire's validity and reliability, and there were no problems with the actual test.

## Measurements

### Mindfulness

The Level of mindfulness Scale was adapted from the Adolescent Level of mindfulness Scale developed by Greco et al. [33]. The original scale, which assesses a person's level of mindfulness, has 10 items with a 5-point Liker scale that range from "never" to "always." (e.g., "I have some bad thoughts and I don't think I should have them "). The scale is reverse scored, and the higher the total score, the higher the level of mindfulness. The internal consistency coefficient of the scale in this study, Cronbach's α was 0.91.

### Psychological resilience

The Psychological Resilience Scale developed by Block and Kreman [34] was used. The original measure consists of 14 items (e.g., "Most people I've met are cute") and is graded on a range of 1 to 4 points (1 = "not at all", 4 = "completely"). and the components were added up to create the entire scale score.The greater the total score, the more psychological resilience was shown to exist. The scale's internal consistency coefficient in the current investigation was 0.92.

### Depression

The Depression Self-Rating Scale used in this study was developed by W.K. Zung in 1965 [35]. The scale has 20 items (e.g., "I think others would live better if I died") and uses a 4-point Likert scale (1 = "no or little time", 4 = "constantly"), half of the items were reverse scored, with higher scores indicating more severe depression. The internal consistency coefficient of the questionnaire was 0.84 in the actual test.

### Statistical methods

Descriptive statistics and a correlation analysis of the data were performed using SPSS 24.0., and PROCESS 3.4 was used for mediating and moderating effect analysis.

## Results

### Common method bias and test

Because the data for this study were gathered by questionnaire using random sampling, there is a possibility of common data bias, and a bias test is required. For the test, we employed the Harman single-factor technique and discovered that there were ten eigenvalues greater than one. The first component explained 23.52% of the variation, which was less than the crucial criterion of 40%, and there was no common technique bias.

### Descriptive statistics of each variable

The descriptive statistics and correlations of each variable are shown in Table 1 mindfulness were significantly positively correlated with psychological resilience (*r* = 0.22, *p* < 0.01) and significantly negatively correlated with depression among college students (*r* = -0.36, *p* < 0.01); psychological resilience was significantly negatively correlated with depression among college students (*r* = -0.45, *p* < 0.01).

**Table 1:** Descriptive statistics and correlation analysis

	1	2	3	4	5	M	SD
1.Mindfulness	1					36.73	7.31
2.Psychological resilience	0.22^**^	1				38.70	8.06
3.Depression	-0.36^**^	-0.45^**^	1			40.72	8.38
4.Gender	-0.23^**^	-0.13^**^	0.02^***^	1		1.41	0.49
5.Grade	0.11^**^	-0.02	-0.14^**^	0.05^*^	1	1.96	1.10

^*^p < 0.05

^**^p < 0.01

^***^p < 0.001

### Mediation test of psychological resilience

The mediating effect of psychological resilience on mindfulness on depressed mood in college students was analyzed while controlling for gender and grade variables. The data were processed using PROCESS model 4, and the results showed (in Table 2) that mindfulness had a significant negative effect on depression in college students(*β* = -0.37, *p* < 0.001); After putting in the mediating variable psychological resilience, mindfulness still had a significant positive effect on psychological resilience (*β* = 0.21, *p* < 0.001) and considerably and negatively influenced collegiate depression (*β* = -0.40, *p* < 0.001). Psychological resilience significantly and negatively influenced depression among college students (*β* = -0.29, *p* < 0.001). The results of the mediation analysis showed that the mediation effect of psychological resilience in mindfulness on depressed mood of college students held with a mediation effect value of 0.08 and with a mediation effect of 22.22%, its 95% Bootstrap confidence interval was [-0.12,-0.05].

**Table 2:** Analysis of mediating effects of psychological resilience

Variables		Overall fit index			95%CI			
Result	Predictor	*R*	*R*^*2*^	*F*	*β*	LLCI	ULCI	t
Depression	Sex	0.37	0.14	48.69^***^	-0.05	-0.12	0.01	-1.73
	Grade				-0.04	-0.10	0.02	-1.45
	Mindfulness				-0.37	-0.43	-0.31	-11.71^***^
Psychological resilience	Sex	0.25	0.06	19.80^***^	-0.09	-0.15	-0.02	-2.66^**^
	Grade				-0.06	-0.12	0.01	-1.78
	Mindfulness				0.21	0.14	0.27	6.27^***^
Depression	Sex	0.54	0.29	93.80^***^	-0.09	-0.15	-0.03	-3.11^**^
	Grade				-0.07	-0.12	-0.01	-2.41^*^
	Mindfulness				-0.40	-0.46	-0.35	-14.08^***^
	Psychological resilience				-0.29	-0.34	-0.23	-9.79^***^

^*^p < 0.05

^**^p < 0.01

^***^p < 0.001

### Tests for moderating effects of gender

**Fig. 2:**
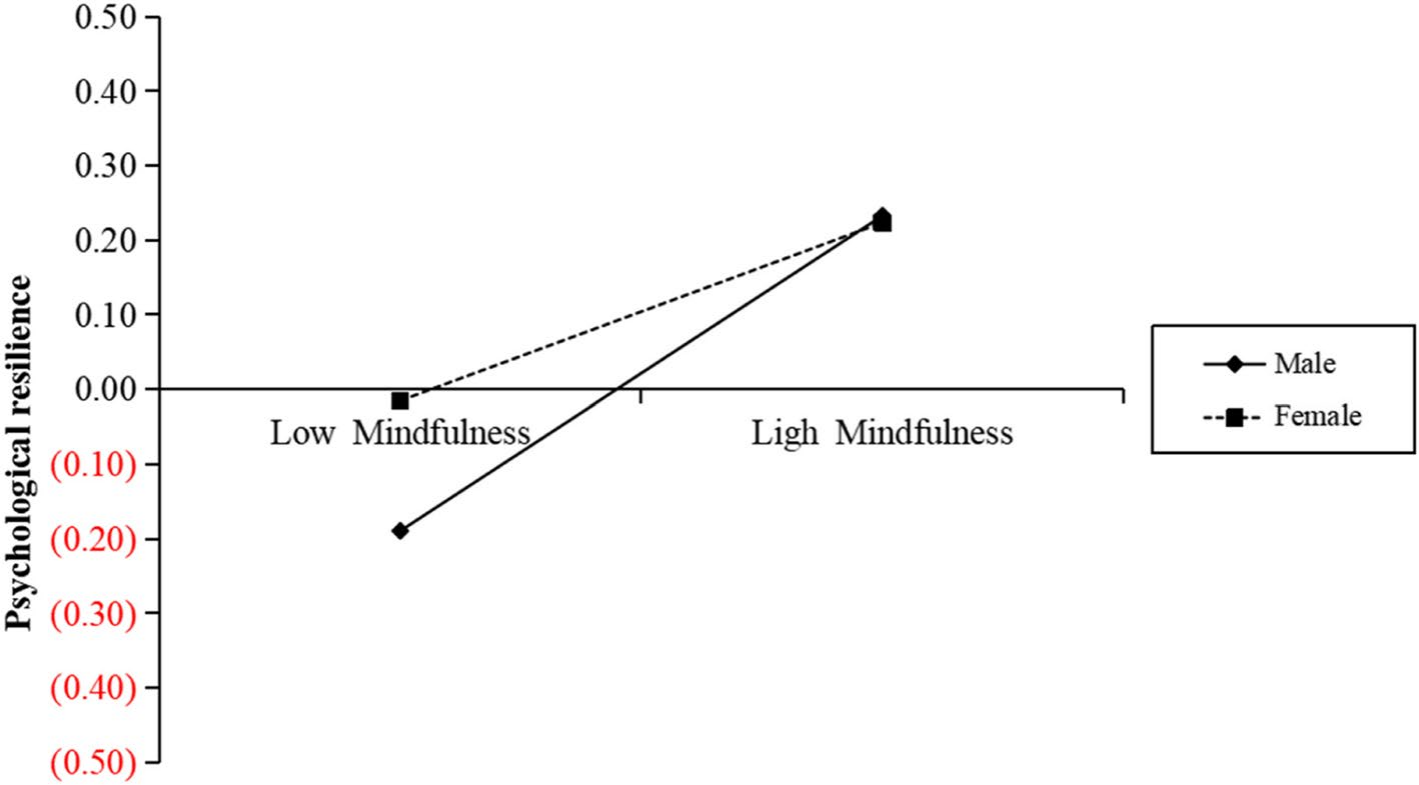
Graph showing how gender influences psychological resilience and mindfulness

The variables were standardized to test whether gender moderated the relationship between mindfulness, psychological resilience, and depressed mood among college students, and the data were processed using Process model 7. The findings revealed (see Table 3) that mindfulness negatively predicted college students' depressed mood significantly (*β* = -0.21, *p* < 0.001) and positively predicted psychological resilience (*β* = 0.22, *p* < 0.001). The product term of mindfulness and gender positively predicted psychological resilience significantly (*β* = 0.09, *p* < 0.01), demonstrating that gender has a moderating effect on the impact of mindfulness on psychological resilience. To further understand the gender difference of mindfulness on psychological resilience, a simple slope analysis was conducted (as shown in Fig. 2). The results showed that when the gender was male, the level of mindfulness moderated psychological resilience significantly (simple slope = 0.22,* t* = 4.92, *p* < 0.001); when the gender was female, the level of mindfulness also moderated psychological resilience significantly (simple slope = 0.31, *t* = 2.83, *p* < 0.01). Compared to male students, the level of mindfulness predicted stronger psychological resilience in female students, which means that female college students' psychological resilience increases as their mindfulness level increases.

**Table 3:** Mediated model tests with moderation

Variables		Overall fit index			95%CI			
Result	Predictor	*R*	*R*^*2*^	*F*	*β*	LLCI	ULCI	*t*
Psychological resilience	Grade	0.26	0.07	16.84^***^	-0.06	-0.12	0.01	-1.79
	Mindfulness				0.22	0.15	0.28	6.59^***^
	Sex				-0.08	-0.14	-0.01	-2.39
	Mindfulness^*^ Sex				0.09	0.03	0.16	2.74^**^
Depression	Grade	0.53	0.28	120.71^***^	-0.07	-0.13	-0.02	-2.61^**^
	Mindfulness				-0.21	-0.32	-0.21	-9.29^***^
	Psychological resilience				-0.39	-0.45	-0.34	-13.80^***^

^*^p < 0.05

^**^p < 0.01

^***^p < 0.001

## Discussion

This study examined the process by which mindfulness affects college students' depressed mood and its mechanism of action, as well as the mediating roles played by psychological resilience and gender. The study's findings help us better grasp the connection between mindfulness and depressed mood of college students and its internal mechanism of action.

This study's findings, which are consistent with earlier studies, indicate that mindfulness has a predictive effect on depressed mood among college students [36]. In other words, the higher the level of mindfulness, the lower the depressive mood of college students will be. The reason for this is that, as a conscious awareness, mindfulness training enhances college students' attention and cognitive functions, avoiding falling into rumination, negative thinking [37] and paying more attention to positive stimuli [38]. Positive attention leads to increased emotional regulation in individuals and college students are less likely to become depressed. This suggests that in order to present an empirical basis for the detection and treatment of depression in college students, focus should be directed to the practice of positive attention levels in students.

Through the mediating role of psychological resilience, mediation analysis demonstrated that mindfulness can also affect college students' depression. Previous researchers have also concluded that practicing mindfulness can strengthen people's capacity for activity motivation, regulation, and cognition as well as their psychological resistance to stress [24]. As a psychological protective factor, psychological resilience is indeed able to recover individuals from bad moods quickly [39]. When faced with a difficult situation, people who have greater psychological resilience are more likely to overcome negative emotions while maintaining more positive, objective, and optimistic psychological qualities [40], and to avoid depression, reduce de-automation and avoidance behaviors, and quickly adapt to their environment. Because this study discovered a function for psychological resilience in moderating the relationship between mindfulness and depression among college students, practice implies increasing the amount of psychological resilience. Strengthen the external protective support for college students, such as social support from family and school [41, 42], while concentrating on the development of college students themselves, such as enhancing self-awareness, self-control, and self-confidence [43, 44], to create a benign external atmosphere. Therefore, as a school administrator, when intervening in the psychological problems of college students, we should not only concentrate on the training of mindfulness level of college students, but also on function of "bridge" of psychological resilience, so as to reduce the generation of depressive mood.

Furthermore, this study found that the first half of the mediating effect between mindfulness on depressed mood among college students was moderated by gender. Although the level of mindfulness significantly predicted psychological resilience in both male and female university students, yet compared with male students, this study finds that mindfulness exert a more apparent influence on the psychological resilience of female students. Gender could moderate the influence of psychological resilience on depression. The mindfulness Stress Model theory suggests that after positive cognitive appraisal, individuals' attention spans expand, reconstructing their perceptions of stressful events and enhancing levels of psychological resilience [45]. Girls are therefore more likely to benefit from mindfulness traits. It has also been shown that a group of adolescents who underwent a 6-week positive meditation intervention had a more significant increase in positive emotions in girls compared to boys [28], which reinforces the gender differences in the effects of positive meditation interventions. The psychological resilience of female university students is more influenced by the effect of mindfulness level compared to male students, and the reason for this is that both females and males may show improvement in positive emotions after training in mindfulness [46], but females are significantly higher than males in terms of emotional self-acceptance and a larger cognitive range [47]. Therefore, when individuals face stress, female college students' mindfulness practices are a more powerfully beneficial predictor of psychological resilience. This result enlightens educators to conduct different strategies of mindfulness training according to gender differences and the different personal and physiological characteristics of male and female college students, which will help to enhance the level of psychological resilience and thus more effectively prevent and intervene in depression among college students.

### Implications and shortcomings of the study

This study found the effects of mindfulness on college students' depression, as well as the mediating role of psychological resilience and the moderating role of gender through a survey study of 936 current college students. Introducing the related theory of psychological elasticity to the field of college students' depression further expands the theory of college students' depressed mood. On the one hand, it helps to guide college students to strengthen the training of mindfulness traits to alleviate depression; on the other hand, school administrations can appropriately offer courses on mindfulness training, emphasize the cultivation of psychological resilience, and provide different support for male and female college students to alleviate depression among college students.

This study also has certain shortcomings. First, the research methodology used a self-assessment model of college students to collect information from questionnaires, and subjective reports may have errors, so multiple sources of data collection can be considered in the future. Second, this study used a cross-sectional study, but it is not possible to predict the long-term validity of the results over a period of time, and a longitudinal study could be considered. In addition, college students' depressed mood is influenced by a variety of factors, and there are many other factors that affect the mediating or moderating variables, so more factors can be explored in depth in the future. Finally the sampling range can be expanded so that the sample can be more representative.

## Conclusion

This study investigated the underlying mechanisms of the association between mindfulness and college student depression and constructed a structural model of mindfulness, psychological resilience, gender, and college student depression. This study suggests that mindfulness may not only have a direct effect on college students' depression, but may also have an indirect effect on depression through psychological resilience. At the same time, gender plays a moderating role in the direct predictive effect of mindfulness on psychological resilience; in other words, mindfulness has a greater effect on psychological resilience in female college students.

## Data Availability

The authors will provide the original data set that underlies the conclusions of this study without reservation.
